# Assessing the Equivalence of Paper, Mobile Phone, and Tablet Survey Responses at a Community Mental Health Center Using Equivalent Halves of a ‘Gold-Standard’ Depression Item Bank

**DOI:** 10.2196/mental.6805

**Published:** 2017-09-06

**Authors:** Benjamin B Brodey, Nicole L Gonzalez, Kathryn Ann Elkin, W Jordan Sasiela, Inger S Brodey

**Affiliations:** ^1^ TeleSage, Inc. Chapel Hill, NC United States

**Keywords:** mobile phone, tablet, PROMIS, depression, item response theory, outcomes tracking, PORTAL, TeleSage, behavioral health, special issue on computing and mental health

## Abstract

**Background:**

The computerized administration of self-report psychiatric diagnostic and outcomes assessments has risen in popularity. If results are similar enough across different administration modalities, then new administration technologies can be used interchangeably and the choice of technology can be based on other factors, such as convenience in the study design. An assessment based on item response theory (IRT), such as the Patient-Reported Outcomes Measurement Information System (PROMIS) depression item bank, offers new possibilities for assessing the effect of technology choice upon results.

**Objective:**

To create equivalent halves of the PROMIS depression item bank and to use these halves to compare survey responses and user satisfaction among administration modalities—paper, mobile phone, or tablet—with a community mental health care population.

**Methods:**

The 28 PROMIS depression items were divided into 2 halves based on content and simulations with an established PROMIS response data set. A total of 129 participants were recruited from an outpatient public sector mental health clinic based in Memphis. All participants took both nonoverlapping halves of the PROMIS IRT-based depression items (Part A and Part B): once using paper and pencil, and once using either a mobile phone or tablet. An 8-cell randomization was done on technology used, order of technologies used, and order of PROMIS Parts A and B. Both Parts A and B were administered as fixed-length assessments and both were scored using published PROMIS IRT parameters and algorithms.

**Results:**

All 129 participants received either Part A or B via paper assessment. Participants were also administered the opposite assessment, 63 using a mobile phone and 66 using a tablet. There was no significant difference in item response scores for Part A versus B. All 3 of the technologies yielded essentially identical assessment results and equivalent satisfaction levels.

**Conclusions:**

Our findings show that the PROMIS depression assessment can be divided into 2 equivalent halves, with the potential to simplify future experimental methodologies. Among community mental health care recipients, the PROMIS items function similarly whether administered via paper, tablet, or mobile phone. User satisfaction across modalities was also similar. Because paper, tablet, and mobile phone administrations yielded similar results, the choice of technology should be based on factors such as convenience and can even be changed during a study without adversely affecting the comparability of results.

## Introduction

As Internet and electronic survey administration technologies have shown many advantages and benefits relative to paper forms, computerized administrations of diagnostic and outcome measures have grown in popularity [1]. Recent studies have shown that participants often prefer the electronic version of an assessment to traditional paper surveys [2,3]. Additionally, electronic data entry has been shown to minimize errors that occur during traditional paper data collection [1,4]. In many situations, however, patients and research participants alternate between paper and electronic data collection (EDC) mediums as needed, such as when certain parts of a facility differ in regards to wireless connectivity, or when it is unknown what device an end-user may use to complete a survey sent as a link in an email. In cases such as these, researchers need to know whether the administration technology meaningfully affects results and whether these technologies can be used interchangeably within a single study. If administration method does not significantly impact assessment results and user satisfaction, the least expensive, most user-friendly, or most convenient form of administration technology can be employed without risk of jeopardizing assessment validity.

Several authors have provided evidence that the results of assessments administered via EDC methods are equivalent to results of those administered via the traditional paper-and-pencil method [1-3,5-9]. Additionally, a 2008 meta-analysis found equivalence between paper- and computer-administered self-report assessments [10]. Similar to prior research, this study investigates whether results of self-report assessment differ based on mode of administration; however, this study improves upon past research in several ways.

First, many studies have used a test-retest design, using the same items or instrument for both assessment periods [5,7-9]. This can be problematic because if the same items are administered sequentially, results may be impacted by a lingering memory effect. Furthermore, when time-delay methods are used to decrease this memory effect, if is not possible to determine whether changes in response are due to changes in modality or changes in symptoms over time. This study provides a method for overcoming these challenges.

A second way that this study improves upon past research in the field is in regard to psychometric equivalence versus face validity equivalence. Previous research has been done using the Patient-Reported Outcomes Measurement Information System (PROMIS) depression item bank (the same items that are used in the current study), which demonstrated the psychometric reliability and validity of these items [2]. While it is true that any 2 sets of items drawn at random from the PROMIS depression item bank should be psychometrically equivalent, clinicians rely on constellations of symptoms to diagnose and understand psychiatric disorders. Thus, from a clinical perspective, it is necessary to have equivalence on the symptom level, as well as psychometrically.

To address both of these concerns, this study created 2 psychometrically equivalent halves of the PROMIS depression item bank. The 2 halves (called Form A and Form B) had no overlapping questions, which eliminated the risk of lingering memory effects within participants. Additionally, to the greatest extent possible, the halves were created to assess similar depression symptoms, which is crucial for an assessment to have clinical significance. We hypothesize that the within-person validity of assessment will be similar across administration modality.

## Methods

### Item Set Generation

The PROMIS depression item bank is a set of 28 self-report items that use a Likert-scale with 5 options that range from “Never” to “Always” indicating how often the patient experiences each symptom [11]. Item response theory (IRT) parameters have been established for the PROMIS items using the graded response model (GRM) [12]. IRT parameters describe the probability of a given response to an item as a function of the respondent’s true standing on a trait or domain (for an overview of IRT and its importance in the field of psychiatry, see Yang and Kao [13]). Thus, IRT allows for estimation of this trait score (theta), and the associated standard error, using any combination or number of items. These PROMIS item parameters and the IRT algorithm were programmed into the TeleSage IRT engine, which runs on the TeleSage data collection platform, called PORTAL. The IRT algorithms were created with assistance from Seung Choi, who also assisted with development of the PROMIS assessment center algorithms [14]. For this study, we chose to use the PROMIS items and parameters due to the rigor that was used in their development and their proven relevance in the field [12].

Dividing the PROMIS depression item bank into 2 nonoverlapping analogous subsets of 14 items created the item sets used in this study. Although a perfect correspondence of content within pairs was not possible, Dr Brodey, a psychiatrist with clinical experience, paired the most similar items together based on criteria from the Diagnostic and Statistical Manual of Mental Disorders, fifth edition (DSM-5). For example, sadness was paired with depression. Sadness and depression are represented by unique PROMIS items but are included in a single DSM-5 criterion [15]. The members of each pair were then divided into Form A and B (see Textboxes 1 and 2). Dividing the PROMIS items based on face validity preserves psychometric equivalence while maximizing clinical equivalence and relevance. The test information curves were derived from the de-identified data set used in the original PROMIS validation [12]. Upon first analysis, one of the item sets provided slightly more information than the other, so one pair was chosen and the 2 items in that pair were switched to the opposite form. The test information curve for the final item sets can be seen in the Results section.

### Data Collection Tool

Electronic health records (EHR) are a ready means of housing and sharing quantitative health information. We used the Health Insurance Portability and Accountability Act of 1996 (HIPAA)-compliant security technologies and an HL7 protocol for the bidirectional exchange of data between the community mental health systems’ EHR and the TeleSage database, via the TeleSage PORTAL.

### Recruitment and Summary of Participants

Following full institutional review board (IRB) approval of this study, participants were recruited via flyers that were posted at an outpatient community mental health center serving severe and persistently mentally ill clients in the Memphis, TN area. Clients were excluded if they were younger than 18 years of age. Participants were advised that they would be paid US$10 in the form of a Target gift certificate regardless of whether or not they completed the study. All 129 participants who began the study completed it. The ages of the participants ranged from 18 to 72 years with an average of 43 years. The participants were more often African-American (109/129, 84.5%), non-Hispanic (123/129, 95.3%), and female (83/129, 64.3%). This is representative of the public sector population served by the clinic used in this study. The demographic characteristics in mobile phone and tablet groups were very similar across age, sex, race, and ethnicity (Table 1).

**Division of Patient-Reported Outcomes Measurement Information System depression bank items into Form A.**

Question text:

- I felt hopeless.
- I felt unhappy.
- I felt sad.
- I felt guilty.
- I withdrew from other people.
- I felt like a failure.
- I felt discouraged about the future.
- I felt ignored by people.
- I found that things in my life were overwhelming.
- I felt that my life was empty.
- I felt disappointed in myself.
- I had trouble making decisions.
- I felt that I was not needed.
- I felt worthless.

**Division of Patient-Reported Outcomes Measurement Information System depression bank items into Form B.**

Question text:

- I felt I had no reason for living.
- I felt that nothing could cheer me up.
- I felt depressed.
- I felt that I was to blame for things.
- I had trouble feeling close to people.
- I felt that I was not as good as other people.
- I felt that I had nothing to look forward to.
- I felt lonely.
- I felt emotionally exhausted.
- I felt that nothing was interesting.
- I felt worthless.
- I felt pessimistic.
- I felt that I wanted to give up on everything.
- I felt upset for no reason.

**Table 1:** Demographics of the full sample and of the mobile phone and tablet administration groups^a^ (see Multimedia Appendix 1).

Demographics		Full Sample (N=129)	Mobile phone (N=63)	Tablet (N=66)
Age; mean (standard deviation)		43 (12)	43 (11.28)	44 (12.63)
**Sex; N (%)**				
	Female	83 (65)	41 (65)	42 (64)
	Male	45 (35)	22 (35)	23 (36)
**Race; N (%)**				
	Asian	1 (1)	1 (2)	0 (0)
	African-American	109 (86)	52 (83)	57 (86)
	Caucasian	17 (13)	9 (14)	8 (12)
**Ethnicity; N (%)**				
	Non-Hispanic	123 (98)	59 (98)	64 (98)
	Hispanic	2 (2)	1 (2)	1 (2)

^a^Missing values: sex (1 in tablet group), race (1 in mobile phone group, 1 in tablet group), and ethnicity (3 in mobile phone group, 1 in tablet group)

### Assessment Modality Assignment and Administration

HIPAA standards were maintained throughout the data collection process. Participants were divided into an 8-cell randomization and independently randomized into groups based on (1) modality of the electronic assessment administration (mobile phone vs tablet), (2) order of assessment modality (paper first vs electronic first), and (3) order of assessment subset presentation (Form A first vs Form B first). All modalities of the surveys were self-administered. The study coordinator at the clinical site provided paper forms, and the electronic assessments were provided via the Internet (using clinic Wi-Fi and a Samsung tablet [n=63] or mobile phone [n=66]) via the TeleSage PORTAL. The site study coordinator entered all the paper surveys into the TeleSage PORTAL by using the rapid data entry interface. TeleSage obtained demographic data on each participant by automatically matching participants with their NetSmart EHR. The PORTAL system integrated with the clinic EHR, allowing the direct importation of demographics from the EHR and direct export of the clinical report to the EHR. The demographic data for each individual, including age in years, sex, race, and ethnicity, were prepopulated into the study assessments without error via the PORTAL system. Assessment reports were generated and exported to the EHR in real time. After completing both survey modalities, 38 consecutive participants filled out a short satisfaction survey (on paper) regarding the technologies they used. The survey asked participants to compare their satisfaction with the paper survey versus the electronic survey, and it asked about satisfaction with the specific electronic modality they used. All questions used a 5-point Likert-scale format of “Strongly Agree” to “Strongly Disagree.” Participants also completed survey items that asked about their technology ownership and usage.

### Statistical Analysis

Scores are evaluated on a theta scale, based on PROMIS community norms and defined from −4.0 to 4.0, where 0 is the mean, and positive scores indicate depression. The PORTAL’s IRT module estimated a trait score (theta) in real time for each of the 2 surveys taken by each individual using the GRM and the maximum likelihood estimation calculation method [12]. The theta scores were subsequently analyzed using mixed-effects models with a random intercept, which allowed for variance in the severity of depression symptoms reported by participants. Additionally, participants were repeated in the data set, which allowed the model to take within-subject dependencies across administrations into account. Fixed-effects predictors included modality (paper, mobile phone, tablet), item set (Form A or B), and the interaction between modality and item set.

To gain a more intuitive understanding of trends seen in the data, *t* tests were also performed. While *t* tests do not take into account all dependencies in the data, they do allow for a more direct comparison of within-subject variation (repeated measures *t* tests of Form A vs B, paper vs mobile phone, and paper vs tablet) and between-subject variation (an independent groups *t* test of mobile phone versus tablet; Multimedia Appendix 1).

## Results

### Item Set Generation

**Figure 1:**
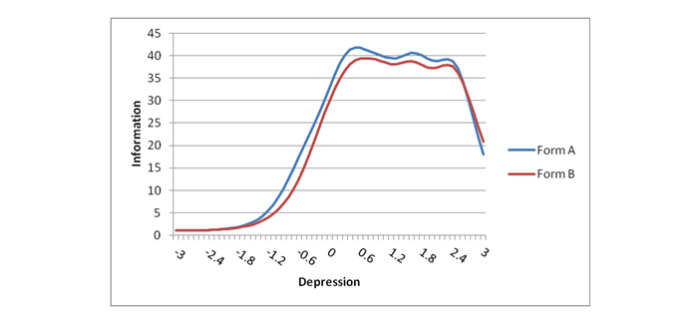
Item response theory test information plot for Forms A and B (see Multimedia Appendix 1).

Using the methodology described previously, it was possible to create 2 psychometrically equivalent halves of the PROMIS depression item bank. Figure 1 depicts the test information plots for Form A and B, based on item data from the original PROMIS validation [12]. Table 2 shows a summary of the IRT scale (theta) scores, overall, and based on variables of interest (Multimedia Appendix 1).

**Table 2:** Summary of item response theory scale scores, overall and by variables of interest (see Multimedia Appendix 1).

Condition		N	Mean	Standard deviation
Overall		258	0.91	0.98
**Modality**				
	Paper	129	0.91	0.87
	Mobile phone	63	0.89	1.04
	Tablet	66	0.93	1.11
**Form**				
	A	129	0.92	0.91
	B	129	0.90	1.04
**Modality × Form**				
	Paper × A	65	0.85	0.92
	Paper × B	64	0.98	0.82
	Mobile phone × A	32	0.93	1.02
	Mobile phone × B	31	0.85	1.08
	Tablet × A	32	1.05	0.79
	Table × B	34	0.81	1.34

### Statistical Analyses

Dr RJ Wirth, of Vector Psychometric Group, completed all statistical analyses. The wording of this section was taken from Dr Wirth’s report (see Multimedia Appendix 1). For the full data analyses, the first model included modality, form, and the modality-by-form interaction as predictors. Results showed a statistically nonsignificant interaction, indicating that the difference between forms did not depend on modality; *F*_2,125_=0.44, *P*=.64. For parsimony, the nonsignificant modality-by-form interaction was dropped and a second, main effects only model was estimated using modality and form as predictors. Results from this main-effects model demonstrated that there was not a statistically significant effect of either form, *F*_1,126_=0.06, *P*=.81, or modality, *F*_2,126_=0.16, *P*=.85 on the provided IRT scale scores for depression.

Similar results were obtained for the model using only data from unflagged observations, which resulted in the removal of 12 subjects for a reduced N of 117. Initial model results showed a statistically nonsignificant interaction, indicating that the difference between forms did not depend on modality; *F*_2,113_=0.39, *P*=.68. For parsimony, the nonsignificant modality-by-form interaction was dropped and a second model was estimated with only modality and form as main effect predictors. Results from this model again demonstrated that there were no statistically significant effects due to either form, *F*_1,114_=0.15, *P*=.70, or modality, *F*_2,114_=0.23, *P*=.79 on depression scores.

The data was also analyzed using *t* tests. While *t* tests do not model as many dependencies in the data, they are often easier to interpret. The results of the repeated-measures *t* tests (comparing the means of Form A and B, as well as paper vs mobile phone scores and paper vs tablet scores) are shown in Table 3. The results of the independent groups *t* test (comparing mobile phone vs table scores) are shown in Table 4. The results of the *t* tests support the general findings of the previously reported analysis of the variance; no statistically significant differences were found among any of the modality comparisons or across forms.

### Post-Assessment Satisfaction and Technology Usage and Experience Survey

After completing both the electronic and the paper assessments, 38 participants in our study received a paper satisfaction survey. Of the mobile phone and tablet groups, 62% (39/63) and 61% (40/66), respectively, responded that they agreed or strongly agreed with the following statement: “It was easier to read the questions on the mobile phone/tablet (than on the paper form).” Of the mobile phone and tablet groups, 61% (38/63) and 72% (48/66), respectively, responded that they disagreed or disagreed strongly with the following statement: “It took me longer to take the survey on the mobile phone/tablet (than on the paper form).” Of the mobile phone and tablet groups, 50% (32/66) and 48% (32/66), respectively, responded that they agreed or strongly agreed with the following statement: “Overall, It was easier to take the survey on the mobile phone/tablet (than on the paper form).” Of the mobile phone and tablet groups, 66% (42/63) and 67% (44/66), respectively, responded that they agreed or strongly agreed with the following statement: “In the future, I would be equally willing to take a survey on paper or using the mobile phone/tablet.” These results indicate that overall, the participants felt that the technologies were largely equivalent.

Analysis of the technology usage and experience survey showed that technology access in the 2 groups was essentially equivalent. Personal computer ownership was 22% (14/63) for the mobile phone group and 18% (12/66) for the tablet group. The mean observed duration for assessment completion on both the mobile phone and tablet was very similar (3.61 and 3.41 minutes, respectively). The mean duration for paper administration was 1.66 minutes. The mean duration of Parts A and B electronic survey administrations were very similar (3.57 and 3.43 minutes, respectively).

**Table 3:** Group descriptives and associated *t* test values for repeated measures planned comparisons (see Multimedia Appendix 1).

Group		N	Mean	Standard deviation	Degrees of freedom	*t*	*P*	Cohen
Form A		129	0.92	0.91				
Form B		129	0.90	1.04				
Difference			0.01	0.59	128	0.25	.80	0.02
**Mobile Phone Group**								
	Paper	63	0.85	0.93				
	Mobile phone	63	0.89	1.04				
	Difference		−0.03	0.66	62	0.42	.68	0.04
**Tablet Group**								
	Paper	66	0.97	0.81				
	Tablet	66	0.93	1.11				
	Difference		0.04	0.53	65	0.68	.50	0.04

^a^Cohen *d* was calculated using original group standard deviations, rather than difference standard deviation [16].

**Table 4:** Group descriptives and *t* test results for the mobile phone versus tablet independent groups comparison (see Multimedia Appendix 1).

Group	N	Mean	Standard deviation	Degrees of freedom	*t*	*P*	Cohen
Mobile phone	63	0.89	1.04				
Tablet	66	0.93	1.11				
Difference		−0.04	1.08	127	−0.2	.84	0.04

## Discussion

### Principal Results

This study found no significant difference between the 2 item sets created from the PROMIS depression item bank; therefore, Forms A and B functioned equivalently in our sample. This suggests that in the future, researchers can administer Forms A and B to the same participant, in the same visit, without results being biased by a memory effect. Future studies could implement the methodology used in this study to assess the equivalence of additional technologies (eg, interactive voice response and smart eye wear [17,18]), or the equivalence of different administration settings (eg, the clinician’s office vs a patient’s home). Additionally, using multiple equivalent item groups may improve methodologies involving regularly repeated longitudinal assessments, by reducing any memory bias.

We also found no significant differences between EDC method and paper, or between mobile phone and tablet. The negligible effect sizes of the differences between assessment modalities suggest that these technologies functioned equivalently within our sample, which is consistent with previous literature [1-3,5-10]. These findings imply that clinicians and researchers can administer the PROMIS depression items to public sector mental health recipients via mobile phone, tablet, or paper, without impacting the reliability of the information gathered from each modality, and can even shift between survey administration technologies during a study without fear of significantly affecting the validity of the survey responses or confounding the study results.

Along with modality and form equivalence, the satisfaction survey reveals that there was no modality (electronic or paper) that participants clearly preferred. This was a surprising finding because the EDC methods took, on average, approximately twice as long as the paper surveys. We do not have clear evidence explaining this variation, but it may be that the EDC modality was relatively novel for many participants, thus it took them extra time to learn how to navigate the electronic surveys. Despite the time difference, a majority of the participants disagreed or disagreed strongly with the statement that it took longer to complete their EDC method. This suggests that patients/participants may not be averse to longer surveys if the surveys are administered electronically.

### Limitations

Many of the recruited clients suffered from schizophrenia. This may have impaired their ability to respond to survey questions. Additionally, this study was conducted with the PROMIS items, which were designed to be short and easy to interpret. Thus, the results might not generalize to more complex question formats.

### Comparison With Prior Work

There are several strengths of the current study that expand upon work done previously. While there has been work done using the PROMIS depression item bank and alternate methods of administration, this may be the first study to use nonoverlapping, equivalent item sets [2]. This methodology could be applied to other instruments in which modality equivalence has been found, to provide greater strength to these studies [3,5,7,8]. One study used 2 different self-report instruments to assess depression and compare modalities, but the authors found significant main effects and interaction effects based on the order in which the 2 instruments were administered [6]. While using 2 different but psychometrically equivalent instruments may have eliminated the risk of memory effect in the previous study, it could have benefitted from the methodology in this study—administering nonoverlapping items from the same assessment (to decrease the effects of administration order) [6].

Additionally, several prior studies have found that participants prefer using an EDC method to a paper survey [2,3,8]. The current study did not have results that are consistent with these studies, suggesting that user preference can change.

### Suggestions for Future Research

Future work should investigate the equivalence of data collected in different settings. With the PORTAL software, clients can easily be administered a survey in their homes via an email or text link (this study’s IRB approval required that all data be gathered within a health care setting). Future research in the public sector mental health care field would benefit from further research of user preference. Finding a modality that most patients are satisfied with could increase both study participation rates and the accuracy of diagnoses, especially if a self-report diagnostic assessment can be administered at home using EDC methods.

### Conclusions

The current study found that, in a population of mental health care recipients, 3 different self-report assessment modalities (mobile phone, tablet, paper) yielded essentially identical assessment results and essentially equivalent satisfaction levels. This suggests that, at least for the PROMIS depression assessment and public sector mental health recipients, the choice of survey administration technology in future studies can be based on cost and convenience. The results may open the way for more accurate technology comparisons among depressed patients.

## Appendix

Multimedia Appendix 1 Statistical report from Dr RJ Wirth.

## Abbreviations

DSM-5: Diagnostic and Statistical Manual of Mental Disorders, fifth edition
EDC: electronic data collection
EHR: electronic health record
GRM: graded response model
HIPAA: Health Insurance Portability and Accountability Act of 1996
IRB: institutional review board
IRT: item response theory
PROMIS: patient-reported outcomes measurement information system
