# Supplementary material for: Assessing the Equivalence of Paper, Mobile Phone, and Tablet Survey Responses at a Community Mental Health Center Using Equivalent Halves of a ‘Gold-Standard’ Depression Item Bank
Source: JMIR Ment Health. 2017 Sep 6;4(3):e36. doi: 10.2196/mental.6805 (PMC5607438; doi:10.2196/mental.6805)
Supplement: Multimedia Appendix 1 [file mental_v4i3e36_app1.pdf]

VECTOR PSYCHOMETRIC GROUP, LLC

PROJECT REPORT

CONFIDENTIAL

Report Title:	Depression score by administration mode and form
Project Title:	PORTAL
Project/Protocol:	N/A
Project Manager:<br>Email:	RJ Wirth, PhD<br>rjwirth@vpgcentral.com
Sponsor or Client:	TeleSage, Inc.
Primary Contact:<br>Email:	Benjamin Brody, MD, MPH<br>bb@telesage.com
Description:	Analyses were conducted to investigate the equivalence of forms and modes of administration (paper, tablet, smartphone) for scores obtained from scales using items from a previously calibrated depression item bank.
Version:	3
Last Updated:	5.4.2016

NOTICE: The information contained in this report is confidential. If you are not an authorized recipient, you are hereby notified that any use, dissemination, distribution, or copying of this report or information contained in this report is prohibited.

If you obtained a copy of this report in error, please immediately notify Vector Psychometric Group, LLC by telephone at 919.768.7142.

Contents

List of Tables 3
Abbreviations 4
Executive Summary 5
Methods 5
Results 5
Conclusions 6
References 7

List of Tables

Table 1. Demographic summary of full sample (N = 129) 8
Table 2. Summary of provided IRT scale scores, overall and by variables of interest 9
Table 3. Group descriptives and t-test results for the Smartphone versus Tablet independent groups comparison 10
Table 4. Group descriptives and associated t-test values for repeated measures planned comparisons 11

Abbreviations

Abbreviation	Definition
ANOVA	Analysis of variance
df	degrees of freedom
IRT	Item response theory
M	Mean
SD	Standard deviation
VPG	Vector Psychometric Group, LLC

Executive Summary

Vector Psychometric Group, LLC (VPG) was contracted by Telesage, Inc. to conduct analysis of provided item response theory (IRT) scale scores for depression to determine the predictive effect, if any, that mode of item administration (paper, tablet, smartphone) and form had on the final scores. A sample of 129 unique subjects was provided, in which each subject completed two visits. Each subject completed a paper administration at one visit and an electronic administration (either on a tablet or smartphone) during the other; forms and mode of administration was based on random assignment. Analysis of variance (ANOVA) models allowing for subject-specific random intercepts were fit to the data as the primary analyses. Results indicated that all scores were equivalent across the forms and modes of administration; that is, neither form, mode, nor their interaction was a statistically significant predictor of the provided depression IRT scale scores. Additional analyses (i.e., t-tests) using simpler models were also conducted to aid in the dissemination of results.

Methods

Data were provided by Telesage for 129 individuals, each of whom had completed two administrations of depression items from the PROMIS item bank. Available demographic characteristics of the sample are presented in Table 1.

In terms of research design, one data collection instance was administered on paper for all subjects, the other administration was via an electronic data collection (EDC) medium, either on a tablet or a smartphone. Additionally, across the two visits, subjects were presented both a Form A and a Form B, which contained non-overlapping subsets of 17 items each from the previously validated PROMIS depression item bank. It is VPGs understanding that the order of form presentation and administration mode were randomly assigned at the subjects' first visit. Electronic device (tablet or smartphone), when warranted by randomization to EDC, was also randomly assigned.

Data were analyzed using mixed effects models with a random intercept, which allowed subjects to vary in their depression levels, and it was specified that subjects were repeated in the dataset to allow the model to account for within-subject dependencies across visits. Visit order was not available for explicitly modeling change over time. Fixed effects predictors included modality (paper, smartphone, tablet), form (Form A or B), and the interaction between modality and form. Analyses were run twice, once using all available subjects and a second omitting observations that had been flagged by TeleSage as questionable due to data irregularities.

Further, to provide more easily disseminated methods and results, several t-test were conducted. Each of these analyses, being limited to comparing only two groups at a time, does fail to model some of the dependencies in the data, but the presentation of group means, SDs, and t-tests is intended more for descriptive purposes and intuitive understanding of the trends seen in the data, rather than as definitive analyses. Specifically, an independent samples t-test was conducted between the smartphone and tablet groups and paired (repeated measures) t-tests were conducted comparing the group means of form A and form B, as well as paper versus smartphone scores, and paper versus tablet scores. Due to the randomization to the two EDC devices, the available sample sizes for these analyses was approximately half of the total sample.

Results

For the full data analyses, the first model included modality, form, and the modality-by-form interaction as predictors. Results showed a statistically non-significant interaction, indicating that the difference between forms did not depend on modality; $F(2,125) = 0.44$, $p = .64$. For parsimony, the

non-significant modality-by-form interaction was dropped and a second, main effects only model was estimated using modality and form as predictors. Results from this main effects model demonstrated that there was not a statistically significant effect of either form, $F(1,126) = 0.06$, $p = 0.81$, or modality, $F(2,126) = 0.16$, $p = 0.85$ on the provided IRT-scale scores for depression.

Similar results were obtained for the model using only data from unflagged observations, which resulted in the removal of 12 subjects for a reduced N of 117. Initial model results showed a statistically non-significant interaction, indicating that the difference between forms did not depend on modality; $F(2,113) = 0.39$, $p = .68$. For parsimony, the non-significant modality-by-form interaction was dropped and a second model was estimated with only modality and form as main effect predictors. Results from this model again demonstrated that there were no statistically significant effects due to either form, $F(1,114) = 0.15$, $p = 0.70$, or modality, $F(2,114) = 0.23$, $p = 0.79$ on depression scores.

The dataset was also analyzed using t-tests to provide what VPG considers a more accessible analysis for non-statistically oriented reviewers. The group means, SDs, and associated t-test values for the previously described comparisons are presented in Tables 3 and 4. As can be seen, and confirming the general findings of the previously reported ANOVAs, no statistically significant differences were found among any of the modality comparisons or across forms.

Conclusions

Overall, analyses supported the contention that the forms were equivalent and that mode of administration played little role in influencing the IRT-based scale scores for depression. The primary analyses that should be referenced in supporting this conclusion are the reported F-test values from the ANOVAs; the reported t-tests analyses provide an incomplete analysis of all dependencies among the data points and were provided primarily for descriptive purposes.

References

Dunlop, W. P., Cortina, J. M., Vaslow, J. B., & Burke, M. J. (1996). Meta-analysis of experiments with matched groups or repeated measures designs. *Psychological Methods, 1,* 170-177.

Table 1. Demographic summary of full sample (N = 129)

Variable	N		M	SD	Min	Max
Age	129		43.12	11.95	18	72
			Percent			
Sex	128					
Female		83	64.84			
Male		45	35.16			
Race	127					
Asian		1	0.79			
African-American		109	85.83			
Caucasian		17	13.39			
Ethnicity	125					
Non-Hispanic		123	98.4			
Hispanic		2	1.6			

Table 2. Summary of provided IRT scale scores, overall and by variables of interest

Grouping		N	M	SD	Min	Max
Overall		258	0.91	0.98	-1.46	3.49
Form						
A		129	0.92	0.91	-1.46	3.49
B		129	0.90	1.04	-1.45	3.17
Modality						
Paper		129	0.91	0.87	-1.46	3.49
Smartphone		63	0.89	1.04	-1.46	3.17
Tablet		66	0.93	1.11	-1.46	3.16
Modality * Form						
Paper	A	65	0.85	0.92	-1.46	3.49
Paper	B	64	0.98	0.82	-1.45	2.54
Smartphone	A	32	0.93	1.02	-1.46	2.37
Smartphone	B	31	0.85	1.08	-1.45	3.17
Tablet	A	32	1.05	0.79	-1.46	2.36
Tablet	B	34	0.81	1.34	-1.45	3.16

Table 3. Group descriptives and t-test results for the Smartphone versus Tablet independent groups comparison

Modality	N	M	SD	df	t-value	p	Cohen's d
Smartphone	63	0.89	1.04				
Tablet	66	0.93	1.11				
Difference		-0.04	1.08	127	-0.2	0.84	0.04

Table 4. Group descriptives and associated t-test values for repeated measures planned comparisons

Level/Comparison	N	M	SD	df	t-value	p	Cohen's d
Form A	129	0.92	0.91				
Form B	129	0.90	1.04				
Difference	129	0.01	0.59	128	0.25	0.80	0.02
Paper	63	0.85	0.93				
Smartphone	63	0.89	1.04				
Difference	63	-0.03	0.66	62	0.42	0.68	0.04
Paper	66	0.97	0.81				
Tablet	66	0.93	1.11				
Difference	66	0.04	0.53	65	0.68	0.50	0.04

Note. Cohen's d calculated using original group SDs, rather than difference SD (Dunlop, Cortina, Vaslow, & Burke, 1996)
